# Association Between Glycated Hemoglobin (HbA1c) Levels in Patients With Iron Deficiency Anemia in a Tertiary Care Hospital in Central India

**DOI:** 10.7759/cureus.66121

**Published:** 2024-08-04

**Authors:** Aniket Patel, Aditya Pundkar, Anshu Agarwal, Charuta Gadkari, Yatrik Vasavada

**Affiliations:** 1 Emergency Medicine, Jawaharlal Nehru Medical College, Datta Meghe Institute of Higher Education and Research, Wardha, IND; 2 Orthopedics, Jawaharlal Nehru Medical College, Datta Meghe Institute of Higher Education and Research, Wardha, IND

**Keywords:** treatment, non-diabetic, association, diabetes, glycated hemoglobin, iron deficiency anemia

## Abstract

Background

Iron deficiency anemia (IDA) and diabetes are prevalent health concerns, especially in regions like India. While previous studies have explored the relationship between glycated hemoglobin (HbA1c) levels and IDA, there is still inconsistency in the findings, particularly in the Indian population. Understanding this association is crucial for accurate diagnosis and management of both conditions.

Materials and methods

A case-control study was conducted at the Department of General Medicine at Acharya Vinoba Bhave Rural Hospital (AVBRH), Wardha, India, from May 2022 to October 2022. A total of 141 non-diabetic patients with IDA (study group) and 141 age- and gender-matched non-anemic controls were included. HbA1c levels were measured at baseline and after three months of IDA treatment. Statistical analysis was performed using SPSS software, including the Kolmogorov-Smirnov test, Chi-square test, Mann-Whitney test, and Pearson correlation coefficient.

Results

In the study group, HbA1c levels significantly increased from a mean of 4.63% at baseline to 5.82% after IDA treatment (p < 0.0001). However, there was no significant correlation between changes in hemoglobin (Hb) levels and HbA1c levels post-correction (r = 0.056, p = 0.510). In addition, all cases and controls were labeled non-diabetic based on a cutoff HbA1c level of 6%. After three months of IDA treatment, 80.85% of cases recovered from IDA.

Conclusion

The study highlights that HbA1c levels are lower in patients with IDA and may increase with the correction of IDA. However, there is no significant direct correlation between IDA correction and HbA1c increase. Therefore, when interpreting HbA1c levels, clinicians must consider the presence of IDA, especially in regions with high prevalence rates of both IDA and diabetes, like India. This understanding can improve management strategies for both conditions, ensuring better patient health outcomes.

## Introduction

Iron deficiency anemia (IDA) and diabetes mellitus are significant public health concerns globally, each with its distinct health implications. IDA, characterized by insufficient iron levels to meet physiological demands, affects individuals of all ages, particularly infants, reproductive-age women, adolescents, and the elderly [1]. It contributes to approximately 50% of cases of anemia worldwide [2]. On the other hand, diabetes mellitus, marked by dysregulation of blood glucose levels, poses a substantial burden on healthcare systems due to its chronic complications and associated comorbidities [3]. One common marker used in diabetes management is glycated hemoglobin (HbA1c), which reflects the average blood glucose levels over the preceding two to three months [4]. Elevated HbA1c levels are indicative of poor glycemic control and are associated with an increased risk of diabetes-related complications [5].

Several studies have investigated the relationship between IDA and HbA1c levels in non-diabetic individuals. Initial investigations reported conflicting findings, with some studies suggesting a correlation between low HbA1c levels and IDA [6,7], while others failed to establish a significant association [8,9]. Furthermore, limited data are available on the Indian population, necessitating further research to elucidate this relationship in this demographic. Recent studies have highlighted the potential impact of correcting IDA on HbA1c levels in non-diabetic individuals. Some findings suggest that treating IDA may lead to a significant increase in HbA1c levels [10,11]. However, the precise mechanisms underlying this relationship remain unclear.

Given the high prevalence of both IDA and diabetes in India, understanding the interplay between these conditions is of paramount importance for clinical practice [12,13]. Accurate interpretation of HbA1c levels is crucial for the appropriate diagnosis and management of diabetes, particularly in populations with a high prevalence of IDA [14,15,16]. In light of these considerations, this study aims to investigate the association between HbA1c levels and IDA in non-diabetic adult patients, focusing on the Indian population. By elucidating this relationship, we aim to contribute to a better understanding of the factors influencing HbA1c levels and inform clinical decision-making in managing both IDA and diabetes.

## Materials and methods

Study design, participants, and setting

This research adopted a case-control study design to explore the relationship between HbA1c levels and IDA in non-diabetic patients. The study was conducted within the Department of General Medicine at Acharya Vinoba Bhave Rural Hospital (AVBRH), a tertiary care facility in Wardha, India, from May 2022 to October 2022. The study enrolled 141 non-diabetic individuals aged 18 to 60 diagnosed with IDA as the case group, alongside 141 age- and gender-matched non-diabetic healthy controls devoid of IDA.

Sample size formula

We determined the sample size for a study with a two-sided confidence level of 95% and a power of 80% using the statistical program Epi Info (CDC, Atlanta, GA, USA, 2011). We considered a 1:1 control-to-case ratio. We targeted detecting an odds ratio (OR) of 0.49. It was expected that 50% of the controls would have the exposure of interest. It was calculated that 32.9% of the instances would have exposure given these conditions. A total of 141 cases and 141 controls made up the required sample size of 282 participants for this investigation.

Sampling technique

Consecutive sampling was done after applying the relevant inclusion and exclusion criteria. 

Inclusion criteria and exclusion criteria

Participants were required to exhibit normal blood sugar levels and have a confirmed diagnosis of IDA, identified through microcytic hypochromic characteristics on peripheral blood film (PBf), mean corpuscular volume (MCV) < 80 fl, mean corpuscular hemoglobin (MCH), < 27 pg/cell, and serum ferritin levels of <22 ng/ml for males and <10 ng/ml for females. Excluded were individuals with diabetes mellitus, chronic illnesses affecting iron metabolism, and pregnant women.

Treatment protocol and laboratory investigations

Patients with IDA received oral iron supplementation (100 mg/day) for three months. Systemic iron therapy was an option for those unable to tolerate oral iron, with the iron requirement calculated using the formula: 2.4 × weight in kg × (Hb deficit (11 - actual Hb)). HbA1c levels were assessed using the fully automated Siemens Dimension RxL Max Immunoassay system (Siemens, Germany) [12]. Serum ferritin levels were measured employing the fully automated Siemens ADVIA Centaur XP immunoassay system. Complete hemogram analysis utilized the Sysmex XP-100 model/MS 95 fully automated blood cell counter (Sysmex, Japan) [13].

Statistical analysis

Data were analyzed utilizing IBM SPSS Statistics for Windows, Version 21.0 (released 2012, IBM Corp., Armonk, NY) [14]. Categorical variables were represented as numbers (n) and percentages (%), while quantitative variables were expressed as mean with standard deviation (mean ± SD) and median with interquartile range (25th to 75th percentiles). The Kolmogorov-Smirnov test determined data normality and appropriate non-parametric tests were applied to the non-normally distributed data. Statistical significance was set at p < 0.05, utilizing the chi-square, Fisher’s exact, Mann-Whitney, and Wilcoxon signed-rank tests.

## Results

A total of 141 patients with IDA aged between 18 and 60 years were included in the study group, and 141 age and gender-matched healthy controls were selected for the control group (Table 1).

**Table 1:** Intragroup HbA1c (%) comparison (using Fisher's exact test and paired t-test) HbA1c: glycosylated hemoglobin

	Pre-correction study group	Post-correction study group	P-value
Non-diabetic (%)	141 (100%)	92 (65.25%)	<0.0001
ADA target (%)	0 (0%)	49 (34.75%)	
Mean ± SD	4.63 ± 0.32	5.82 ± 0.34	<0.0001
Mean difference	1.19 ± 0.48		
Median (25th-75th)	4.6 (4.4-4.9)	5.8 (5.6-6.1)	
Range	4-5.3	5.1-6.8	

**Figure 1:**
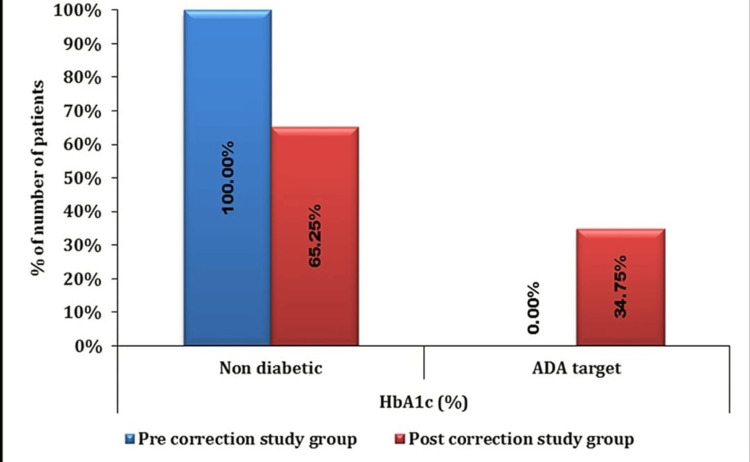
Intragroup HbA1c (%) comparison HbA1c: glycosylated hemoglobin, ADA: American Diabetes Association

The proportion of non-diabetic individuals was significantly higher in the pre-correction study group compared to the post-correction study group (100% vs, 65.25%, respectively) (Figure 1).

Conversely, the proportion of patients achieving the ADA target was significantly lower in the pre-correction study group compared to the post-correction study group (0% vs. 34.75%, respectively), with a p-value of <0.0001. The mean ± SD of HbA1c (%) in the pre-correction study group was 4.63 ± 0.32, which significantly increased to 5.82 ± 0.34 post-correction (p-value < 0.0001) Table 2.

**Table 2:** Correlation of change in post-correction hemoglobin (gm/dl) and HbA1c (%) HbA1c: glycosylated hemoglobin

Variables	Change in HbA1c (%) - post correction
Change in hemoglobin (gm/dl) - post correction	Correlation coefficient: 0.056
	P-value: 0.510

**Figure 2:**
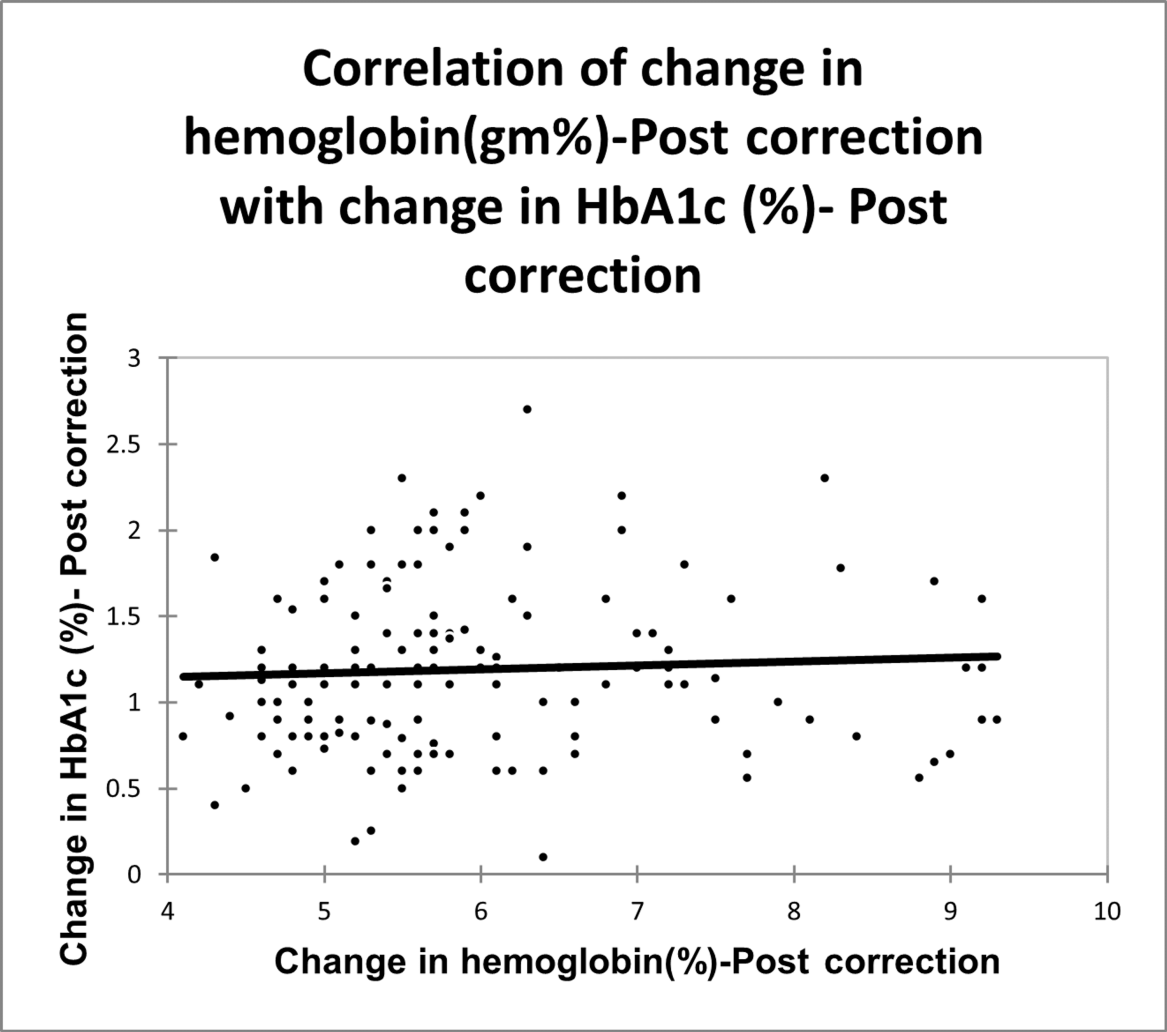
Correlation of change in hemoglobin (gm/dl) post correction with change in HbA1c (%) post correction

No correlation was observed between the change in hemoglobin (Hb) (gm/dl) post-correction and the change in HbA1c (%) post-correction, with a correlation coefficient of 0.056 (Figure 2).

## Discussion

This study adds a great deal to the current discussion on the complex relationship between non-diabetic people's IDA and HbA1c levels. Our findings show a significant drop in HbA1c levels after IDA correction, supporting other studies that have found comparable patterns [7,8]. This observation is significant because it emphasizes how iron status may affect HbA1c measures. It also emphasizes how important it is to take IDA into account when interpreting HbA1c values, particularly in populations where both disorders are common.

The reduction in HbA1c levels following the IDA correction indicates that iron status can have a substantial impact on HbA1c readings in addition to glucose levels [9]. This has significant ramifications for clinical practice, especially in areas where iron deficiency anemia and diabetes are both highly prevalent. For example, in India, where diabetes mellitus and IDA are major public health concerns, the possibility that IDA would influence HbA1c measurements could result in incorrect diabetes diagnosis or treatment [10]. Therefore, in order to prevent incorrect diagnoses and improper treatment plans, clinicians should proceed with caution and take into account the iron status of their patients when interpreting HbA1c values [11,12]. The lack of a significant association between changes in Hb levels and HbA1c levels after IDA correction is one of the main conclusions of our study. This implies that variables other than only Hb concentrations could affect HbA1c values in people with IDA [12]. Because HbA1c control is complex, more research into the underlying mechanisms causing these changes is necessary. For instance, in the setting of IDA [13,14], it is suggested that alterations in erythrocyte turnover, longevity, and glycation kinetics may be responsible for variances in HbA1c levels. A low iron level impacts erythropoiesis and glycated Hb synthesis, possibly modifying the glycation process through modifications in red blood cell (RBC) turnover and survival rates. Furthermore, the effect of iron deficiency on HbA1c levels might differ from person to person depending on a number of variables, including food consumption, iron storage, genetic predisposition, and the degree of anemia [15,16].

Personalized therapy and diagnostic procedures require an understanding of these individual variances. More study is necessary since the exact processes by which iron deficiency affects HbA1c are not entirely understood. For example, more research is needed to fully understand how altered erythrocyte metabolism and possible oxidative stress affect HbA1c levels in IDA patients. Our research supports earlier research showing a correlation between IDA and reduced HbA1c levels; one study found that iron supplementation resulted in a mean HbA1c drop of 0.57% [17,18,19]. Furthermore, our research highlights the necessity of thorough screening and diagnostic approaches that consider the intricate interactions among iron status, glycemic management, and HbA1c levels. In patients with unexplained HbA1c fluctuation, clinicians ought to think about doing routine IDA screening, particularly in areas where iron deficiency is highly prevalent. For a more precise picture of a patient's glycemic condition, this could involve measuring serum ferritin levels, transferrin saturation, and other iron indicators in addition to HbA1c. Public health campaigns should also concentrate on treating the combined burden of iron deficiency anemia and diabetes. Misdiagnosis and incorrect management can be decreased with the use of educational programs that increase awareness about the possible impact of IDA on HbA1c levels.

To sum up, our research contributes to the increasing amount of data highlighting the important impact of iron deficiency anemia on HbA1c levels in people without diabetes. By stressing the need to take IDA into account when interpreting HbA1c results, we draw attention to the significance of a patient-centered approach that incorporates iron status evaluations into regular diabetes therapy. This strategy will guarantee suitable and customized treatment plans, which will not only improve diagnostic accuracy but also improve overall patient results. In order to create tailored therapies that address the complex nature of HbA1c regulation in the context of iron deficiency, more study is necessary to clarify the particular processes at play.

## Conclusions

Our study sheds light on the intricate relationship between HbA1c levels and IDA in individuals without diabetes. We discovered that IDA correction led to a significant reduction in HbA1c levels, indicating the influence of iron status on HbA1c measurements that are utilized to assess long-term glycemic control. Interestingly, after IDA correction, there was no significant correlation seen between changes in Hb levels and HbA1c. This suggests that other parameters, such as erythrocyte turnover and red blood cell longevity, may have an impact on HbA1c regulation in IDA. Our results have important implications for clinical practice, particularly in areas like India where IDA and diabetes are prevalent. When evaluating HbA1c findings, clinicians should take iron status into account to prevent misdiagnosing or treating diabetes. In order to improve patient outcomes through the development of more precise diagnostic tools and treatment plans, future research should investigate the mechanisms influencing HbA1c levels in the context of IDA. The combined burden of diabetes and IDA should be addressed by public health campaigns that support integrated approaches that involve treating and preventing iron deficiency and monitoring glycemic control. Our findings highlight the need to interpret HbA1c readings carefully in relation to iron deficiency anemia and the necessity of providing coordinated care for these related disorders.
